# Editorial: Recent Progresses in Amebiasis

**DOI:** 10.3389/fcimb.2019.00247

**Published:** 2019-07-09

**Authors:** Anjan Debnath, Mario Alberto Rodriguez, Serge Ankri

**Affiliations:** ^1^Center for Discovery and Innovation in Parasitic Diseases, Skaggs School of Pharmacy and Pharmaceutical Sciences, University of California, San Diego, La Jolla, CA, United States; ^2^Centro de Investigación y de Estudios Avanzados del Instituto Politécnico Nacional (CINVESTAV-IPN), Mexico City, Mexico; ^3^Department of Molecular Microbiology, Ruth and Bruce Rappaport Faculty of Medicine, Technion, Haifa, Israel

**Keywords:** *Entamoeba histolytica*, adaptation to stress, metabolism, pathogenesis, microbiome, drug discovery and resistance

The aim of this research topic is to provide an overview of our current knowledge on amebiasis. This major neglected tropical disease which is transmitted by the unicellular protozoan parasite *Entamoeba histolytica* accounted for 55,500 deaths and 2.237 million disability-adjusted life years (i.e., the sum of years of life lost and years lived with disability) in 2010 (Turkeltaub et al., 2015). The parasite has two stages in its life cycle in the host: the infective cyst and the invasive trophozoite. About nine out of 10 people who are infected with *E. histolytica* are asymptomatic and in those individuals who develop symptoms, bloody diarrhea (amebic colitis), and liver abscess are the most common symptoms. Although the exact conditions, which trigger the onset of invasive disease, are still unknown, the interaction between the parasite's virulence factors and the host's response contribute to the development of disease (Huston and Petri, 1998). In recent years, significant advances on the cell biology of *Entamoeba* infection have been achieved through the development of new genetic tools to manipulate gene expression in the parasite and through the application of omics tools. Since the publication of the last book on amebiasis five years ago (Nozaki and Bhattacharya, 2015), more than one thousand publications on this subject have been published. This research topic (RT) which collates 29 articles by 167 authors presents the most recent development in this field through original articles and reviews which we introduce here briefly by classifying them according to four sections along the path from drug discovery, cell biology and signaling, regulation of gene expression, and pathogenesis and immunity.

## Drug Discovery

*E. histolytica* is capable of acquiring resistance to amebicidal concentrations of metronidazole (MTZ), the current drug of choice, under laboratory conditions, and this drug resistance has been associated with an increased expression of iron-containing superoxide dismutase and peroxiredoxin (Wassmann et al., 1999). Moreover, partial resistance to MTZ has also been described in some clinical strains of *E. histolytica*, suggesting the emergence of MTZ resistant strains (Bansal et al., 2004; Iyer et al., 2017). Thus, these observations have led to the search for new drugs with targets and modes of action distinct from those of MTZ [for a recent review see (Nagaraja and Ankri, 2019)]. In this RT, three studies based on drug screening have been published. The first one by the Kumar et al. has led to the identification of 1H-1,2,3-triazole-tethered isatin-metronidazole conjugates, which are more efficient against *E. histolytica* and *Giardia lamblia* than MTZ. The second one, from the Mori et al., targets the essential cysteine biosynthetic pathway of the parasite. The authors have described a fungal metabolite pencolide as the first compound that inhibits cysteine synthase and amebic cell growth in a cysteine-dependent manner with relatively low mammalian cytotoxicity. The third one from the Ehrenkaufer et al. identified anisomycin and prodigiosin as drugs able to kill mature cysts and MNZ resistant *E. histolytica*. Another study by the Probst et al. targets farnesyltransferase (FT), the last common enzyme for products derived from the mevalonate pathway. This enzyme is vital for diverse functions, including cell differentiation and growth. The authors found that a synergistic combination of metronidazole and an FT inhibitor lonafarnib offers a promising treatment strategy for amebiasis. Plants and their extracts are currently used to treat gastrointestinal diseases in many different parts of the world (Kelber et al., 2017). The Martínez -Castillo et al. summarizes our current knowledge on the antiamebic properties of flavonoids, a class of antioxidants compounds with variable phenolic structures which are found in many plants and vegetables.

## Cell Biology and Signaling

*E. histolytica* acquires most of its nutrients by phagocytosis of bacteria present in the gut microbiota of the colon and by phagocytosis of host's cells. This essential event for the development of the parasite is directly involved in its pathogenesis. Actin is a fundamental component of the cytoskeleton which is responsible for changes in cell shape and other pivotal processes, including motility, phagocytosis of human cells, and parasite-substrate interactions. In this RT, the Manich et al. describes the 3D structural major divergences of *E. histolytica* actin compared to human actin. The authors also provide new information on the genesis of actin-enriched structures in *E. histolytica*, and on the role of the Arp2/3 actin-nucleation complex in the dynamics of the actin-rich cytoskeleton. Studying changes occurring in the parasite's cytoskeleton during migration may be challenging. A new method from Sierra-López et al. used glass or plastic surfaces covered with a substrate in a “micropatterned grill line.” This method stimulated adhesion, migration, and an efficient formation of different membrane protrusions of *E. histolytica* trophozoites. On the phagocytosis side, the Avalos-Padilla et al. used an epigenetic silencing approach (Bracha et al., 2006) to demonstrate the essential role of Ehvps20 and Ehvps24, two proteins of the endosomal sorting complex required for transport, in erythrophagocytosis. The Rani Iyer et al. gives a glimpse of phagocytosis when *E. histolytica* is incubated with its favorite food, bacteria, isolated from human fecal material. The authors used a rRNA based metagenomic approach to point out that the parasite prefers to phagocytose a few bacterial species including *Lactobacillus ruminus* which was never shown to be associated with *E. histolytica*.

Two articles dealt with encystation of *Entamoeba invadens*. In the first one from the Mi-ichi et al., the authors used flow cytometry analysis to monitor cell differentiation. The second one from the Krishnan and Ghosh describes the formation of multinucleated giant cells which are formed during encystation by repeated cellular fusion with fusion-competent trophozoites. These observations indicate the possibility of *Entamoeba* undergoing sexual or parasexual reproduction.

During the colonization of the host, *E. histolytica* triggers an acute inflammatory process with the release of cytokines, reactive oxygen species (ROS), and nitric oxide (NO) from activated cells of the immune system. ROS and NO have been reported to trigger stress responses. A number of articles in this RT are dealing with stress responses of the parasite: the Nagaraja and Ankri provides a review of all the studies that used omics approaches to study the parasite's response to stresses. The Azuara-Liceaga et al. provides insight into the role of a DNA ligase involved in repairing DNA following oxidative DNA damage. ROS and NO cause programmed cell death (PCD) in *E. histolytica*. The Domínguez-Fernández et al. investigated the role of a calpain-like protein in triggering PCD in the parasite exposed to the aminoglycoside G418. Another work on PCD is provided by the Valle-Solis et al. The authors identified a protein of the CCX family (EhCCX) which is involved in PCD. Interestingly, the overexpression of EhCCX increased the *in vitro* virulence of trophozoites.

The outcome of two *in silico* genome-wide survey analyses performed by the Nakada-Tsukui et al. are described in this RT. The first one involved the identification of phosphatidylinositol (PI) kinases and PI phosphatases. Although it appears that *E. histolytica* possesses 10 PI kinases and 23 PI phosphatases, class II PI 3-kinases, type II PI 4-kinases, type III PI 5-phosphatases, and PI 4P-specific phosphatases are not represented in the parasite. There are several kinases and phosphatases that have the nuclear localization signal suggesting that PI metabolism also has conserved roles related to nuclear functions in *E. histolytica*, as it does in model organisms.

Regulated trafficking and secretion of pathogenic factors (Nakada-Tsukui et al., 2009; Somlata et al., 2017) and cyst wall proteins (Herman et al., 2017) have been extensively studied in this parasite. In a second review, the Das and Nozaki provides *in silico* data supporting the existence of a well-organized lipid transport in *E. histolytica*. It has been suggested that lipids play central roles in parasite growth, proliferation, differentiation, and virulence (Serrano-Luna et al., 2010).

## Regulation of Gene Expression

A number of articles focused on various aspects of RNA processing during the life cycle of *E. histolytica*. The Valdés-Flores et al. offers a review about our current knowledge on the molecular basis for splicing, 3′ end formation and mRNA degradation in ameba. Insights into the mechanism of splicing of AG-dependent and AG-independent transcripts and the post splicing of full length intron circles are provided by the Torres-Cifuentes et al. These events are involved in the regulation of gene expression of different regulatory processes including virulence traits in the parasite. Finally, the Valdés et al. also provided the first characterization of the RNA lariat debranching enzyme (Dbr1). This enzyme hydrolyzes the 2′-5′ linkage in intron lariats, and modulates snRNP recycling during splicing reactions.

It is now well-documented that telomere length affects gene expression even for genes located far up to 1.2 Mb from the telomeres (Robin et al., 2014; Kim and Shay, 2018). In *E. histolytica*, our knowledge about the parasite's telomeres and their role in controlling gene expression is scanty. It is known that *E. histolytica* telomeres are non-conventional and they are formed by long tandem arrays that contain between 1 and 5 tRNA types per repeat unit and STRs which resemble microsatellites (Clark et al., 2006; Tawari et al., 2008). In the work presented by the Rendón-Gandarilla et al., the authors described the first three *E. histolytica* Telomeric Repeat Binding Factors. One of them, EhTRF-like III, forms specific DNA-protein complexes with telomeric related sequences.

## Pathogenesis and Immunity

The mechanisms leading to the interaction of *E. histolytica* with the intestinal epithelium of the host and its colonization have been recently reviewed (Cornick and Chadee, 2017). In a work from the Betanzos et al., the authors proposed that the EhADH adhesin protein altered tight junction of the host epithelium and that it may consequently make epithelial cells more susceptible to other *E. histolytica* effector proteins.

Immune reaction and immune evasion during amebiasis has been recently reviewed. It includes the suppression by the parasite of IFN-γ production, the elimination of immune cells and soluble immune mediators, and the neutralization of the cytotoxic effect of ROS and NOS produced during the inflammatory process (Nakada-Tsukui and Nozaki, 2016). In this RT, a number of amebic factors that regulate the host immune response are described. A new mechanism used by *E. histolytica* to prevent the triggering of inflammation during invasion of the gut via the production of a cyclooxygenase-like protein (EhCox) is described by the Shahi et al. EhCox alters the activity of *E. histolytica* cysteine proteases that are known to trigger inflammation (Hou et al., 2010). The López-Rosas et al. provided evidence that up-regulation of host microRNA-643 by *E. histolytica* promoted apoptosis of human epithelial colon cells. The Gonzalez Rivas et al., described the role of *E. histolytica* calreticulin (*Eh*CRT) which acts as a mitogen. Indeed, *Eh*CRT specifically activated a Th2 cytokine profile during the acute phase of liver abscess and a Th1 profile during the resolution phase of liver abscess. Two articles from the Fonseca et al. and Díaz-Godínez et al. provided insights into the mechanism of induction of neutrophil extracellular traps (NETs) by *E. histolytica*. NETs are formed by DNA fibers decorated with histones and neutrophils constitute a first line of defense against invading pathogens. The authors showed that NETs formation is induced by Raf/MEK/ERK, extracellular calcium and serine-protease activity but does not depend on PKC, TAK1, ROS, NOX2-derived ROS, and PAD4 activity.

Finally, this RT includes a comprehensive review on the role of *Entamoeba gingivalis* in periodontitis by the Bonner et al. *E. gingivalis* shares a number of features with *E. histolytica* including the ability to phagocytose bacteria and human cells.

## Conclusions

Although considered a neglected disease, research on amebiasis continued unabated. Progress on research in amebiasis encompassed studying the life cycle stages and biology of *E. histolytica*, use of modern tools of genomics and metabolomics to analyze the responses of the parasite to different host stimuli and involvement of chemistry to develop new drug leads to control the parasite. Because of the lack of compilation of a comprehensive report on the progress that happened in the last 5 years in this field, our RT on the recent progress in amebiasis is timely. The RT articles covering different aspects of current amebiasis research may provide an important resource to the scientific community involved in the research of protozoan parasites. This may also serve as a guide for developing new areas of research in the field of amebiasis.

## Author Contributions

All authors listed have made a substantial, direct and intellectual contribution to the work, and approved it for publication.

### Conflict of Interest Statement

The authors declare that the research was conducted in the absence of any commercial or financial relationships that could be construed as a potential conflict of interest.
